# Prevalence of residual limb pain and neuromas after upper extremity amputation: a systematic review and meta-analysis

**DOI:** 10.1177/17531934251345368

**Published:** 2025-05-29

**Authors:** Emile B List, Vita Klieverik, Enrico Martin, David D Krijgh, J Henk Coert

**Affiliations:** Department of Plastic and Reconstructive Surgery, University Medical Center Utrecht, The Netherlands

**Keywords:** Amputation, neuroma, residual limb pain, upper extremity

## Abstract

Residual limb pain (RLP) and symptomatic neuromas are common complications of upper extremity amputation. There is disagreement in the literature about their true prevalence. This systematic review and meta-analysis of the prevalence of RLP and symptomatic neuroma after upper extremity amputation identified relevant studies published between 2000 and 2022 in PubMed, Cochrane and Embase. Random-effects meta-analyses of proportions were performed to determine the prevalence of RLP and symptomatic neuromas. Subgroups were identified and analysed. For RLP, the pooled prevalence was 58% (95% CI: 46 to 68). For symptomatic neuromas, the pooled prevalence was 22% (95% CI: 11 to 40). Residual limb pain was reported less frequently in Europe than in the United States. Knowledge and awareness are important for appropriate treatment and prevention.

## Introduction

Residual limb pain (RLP), also known as stump pain, is defined as pain coming from the remaining part of an amputated limb (Hsu and Cohen, 2013). Phantom limb pain is generally different and is characterized as painful sensations from the missing part of the amputated limb. Both pain conditions are common complications following limb amputation (Subedi and Grossberg, 2011). Residual limb pain can result from a variety of somatic and neuropathic causes and is therefore a general term. Somatic RLP can be caused by wound or prosthesis-related problems, osteogenic or vascular dysfunction, and infection (Clarke et al., 2013). Neuropathic RLP can be caused by a neuroma or an entrapped nerve, and it can also occur in patients with complex regional pain syndrome, a condition where no specific affected nerve can be identified as the cause (Clarke et al., 2013).

A neuroma can develop at the severed end of a nerve severed in an amputation. As a result of axonal nerve injury, an inflammatory state and aberrant regeneration culminate in neuroma formation (Hsu and Cohen, 2013). The majority of neuromas are asymptomatic, but some patients experience severe pain. Other physical symptoms include motor or sensory dysfunction, which may present as hypoesthesia, dysesthesia, paresthesia, hyperalgesia and anaesthesia in the affected nerve region (Colloca et al., 2017). The pain is described as sharp and burning, and a positive Tinel's sign can help diagnose a neuroma (Arnold et al., 2019). Individuals with symptomatic neuromas are impaired in their daily activities, which negatively affects their quality of life (Lee and Guyuron, 2015; Ware and Gandek, 1998).

According to our previous research, the prevalence of RLP and symptomatic neuromas in patients after lower extremity amputation is 59% (95% CI: 51 to 67) and 15% (95% CI: 7 to 28), respectively (List et al., 2021). Upper and lower extremity amputations are difficult to compare owing to differences in function, weight bearing, anatomical nerve locations and prosthesis types. Unlike lower extremity amputations, ones in the upper extremity often result from an injury in young and healthy individuals. Lower extremity amputation is more often indicated in the elderly owing to peripheral vascular disease. There is disagreement in the literature about the true prevalence of RLP and symptomatic neuromas after upper extremity amputation, and general practitioners often underestimate the prevalence (Whyte and Niven, 2004). Both conditions are treatable, and greater medical awareness should be increased. This systematic review and meta-analysis provide a comprehensive overview of the literature on the prevalence of RLP and symptomatic neuromas after upper extremity amputation.

## Methods

In October 2022, we systematically searched the PubMed, Cochrane and Embase databases according to the Preferred Reporting Items for Systematic Reviews and Meta-Analyses (Moher et al., 2009). A search string was constructed using the search terms related to ‘amputation’, ‘residual limb pain’ and ‘neuroma’. Appendix S1 provides the exact search syntaxes for each database. Articles were assessed for relevance by three independent researchers. All studies reporting the prevalence of RLP or symptomatic neuroma in patients, aged ≥18 years who had undergone upper extremity amputation, were included. Interventional cohort studies, animal studies, studies published before 2000, articles in languages other than English, Dutch or German, and studies with a sample size smaller than 20 patients or a mean follow-up of less than 6 months were excluded. In addition, studies for which the full text was not available and those with duplicate data were excluded. Studies on SP were interpreted as RLP and identified as such.

### Data extraction

The following data were extracted using a standardized form: title, authors, year of publication, study design, country, sample size, mean age of participants, sex distribution, follow-up time, reason for amputation, level of amputation, method of data collection and prevalence of RLP and symptomatic neuromas. Any uncertainties were resolved by discussion between the authors.

### Quality assessment

The risk of bias of the included studies was assessed using a tool based on a modified version of the Newcastle–Ottawa scale used in other prevalence systematic reviews and meta-analyses (Mata et al., 2015; Rotenstein et al., 2016, 2018; Stang, 2010). Articles were assessed for quality in terms of representativeness, sample size, comparability between respondents and non-respondents, outcome ascertainment and descriptive statistics. Studies were classified as low risk of bias (≥3 points) or high risk of bias (<3 points). The modified Newcastle–Ottawa scoring guide is shown in Appendix S2.

### Statistical analysis

Random-effects meta-analyses of proportions were performed on all included studies to quantify the prevalence of RLP and symptomatic neuromas after upper extremity amputation. Residual limb pain and SP were considered similar and the results were combined under RLP. Forest plots were constructed to visualize heterogeneity between studies. Subgroup analyses were performed using logistic regression analysis with random-effects models. Studies were grouped according to the characteristics age (>50 years vs. ≤50 years), geographic location (Europe vs. United States), reason for amputation, data collection method (interview vs. self-administered questionnaire) and follow-up period (>10 years vs. ≤10 years). In the subgroup analysis of reason for amputation, the prevalence of RLP was compared in studies of trauma patients (including war injuries) with studies of other amputation aetiologies. Subgroup analyses were performed whenever more than 10 studies were included in the meta-analyses, because smaller samples may lead to biased results owing to a higher incidence of false-positive differences (Higgins and Thompson, 2004).

## Results

**Figure 1.**
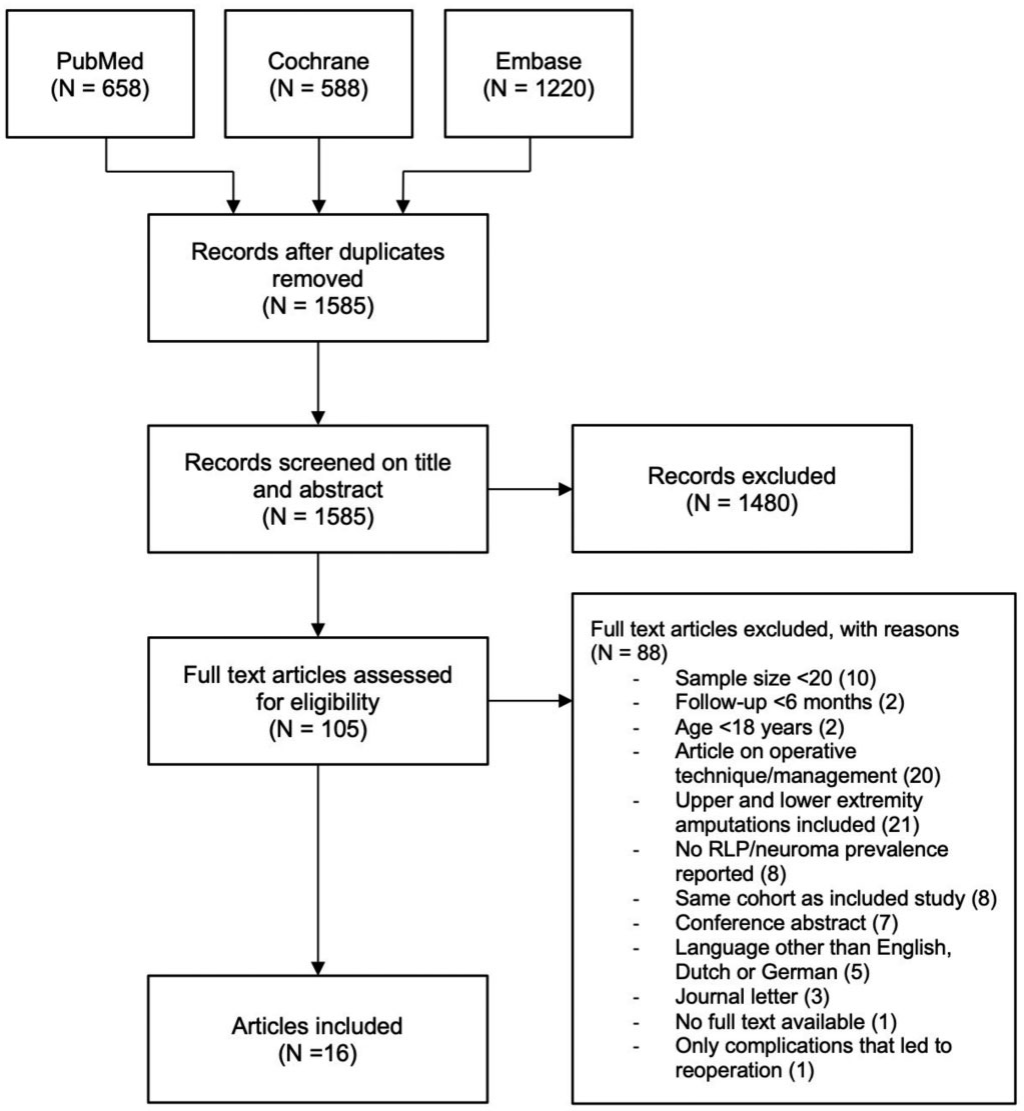
Flowchart systematic literature search.

After removing duplicates, a total of 1585 published articles were identified. After title/abstract screening, 105 full-text articles were assessed, and 16 studies (1777 patients from nine different countries) were included, of which 14 had a cross-sectional design and two a retrospective design (Figure 1). The studies included between 22 and 808 patients, with a mean of 118. Thirteen studies reported the prevalence of RLP, two studies reported the prevalence of symptomatic neuroma and one reported both prevalences. Most studies determined the prevalence by a dichotomous question of presence or absence. Five of the RLP articles also reported the severity using a numeric rating scale, the pooled mean of which was 4.5 on a scale of 0–10, with scores ranging from 3.2 to 5.1. Two studies used medical records to determine prevalence, six used face-to-face or telephone interviews and eight used self-administered questionnaires to collect the data. Five studies reported the specific type of prosthesis used, with the majority of participants using either cosmetic or body-powered prostheses. The quality assessment indicated that seven studies had a high risk of bias, while nine studies had a low risk of bias (Table 1). A detailed overview of the quality assessment of all individual studies is provided in Online Table S1.

**Table 1.** Overview of included studies

Author	Year	Country	Study design	Patients, *N*	Age, years	Men, *N* (%)	Follow-up, years
Bouteille	2022	France	Retrospective	33	Mean: 54.8	27 (82)	Minimum: 0.5
Datta	2004	England	Cross-sectional	60	Mean: 58.1	48 (80)	Mean: 34
De Lange	2022	The Netherlands	Cross-sectional	50	Mean: 51.7	49 (98)	Mean (range): 3.7 (2.1–8.1)
Desmond	2010	Ireland	Cross-sectional	141	Mean (SD): 74.8 (11.4)	139 (99)	Mean (SD): 50.1 (13.5)
Ebrahimzadeh	2006	Iran	Cross-sectional	25	Mean: 41.6	NR	Mean: 18.5
Ephraim^a^	2005	United States	Cross-sectional	100	Mean (SD): 50.3 (13.3)	552 (60)	Median (range): 4 (0–66)
Fraser	2001	England	Cross-sectional	76	Mean (SD): 55 (15)	64 (82)	Mean (SD): 25 (18)
Guo	2019	China	Cross-sectional	22	Mean (SD): 52.3 (8.0)	16 (73)	Mean (SD): 23.7 (13.7)
Hanley	2009	United States	Cross-sectional	104	Mean (SD): 46.9 (14.2)	75 (72)	Median (SD) 7.0 (11.5)
Kooijman	2000	The Netherlands	Cross-sectional	72	Median (IQR): 44.2 (35.2–65)	57 (79)	Median (IQR): 19.1 (6.4–32.1)
Lacoux	2002	Sierra Leone	Cross-sectional	40	Mean (range): 39.3 (16–68)	32 (80)	Mean (range): 22 (10–48)
Lans	2020	United States	Retrospective	142	Mean (SD): 49.0 (15.9)	91 (64)	Mean (SD): 4.8 (4.2)
O'brien	2020	United States	Cross-sectional	55	Median (IQR): 51 (41–61)	39 (71)	Minimum: 0.5
Reiber^b^	2010	United States	Cross-sectional	97	Mean (SD): 44.8 (15.8)	93 (96)	Mean (SD): 21.3 (18.0)
Resnik	2019	United States	Cross-sectional	808	Mean (SD): 63.3 (14.1)	787 (96)	Mean (SD): 31.4 (18.3)
Schley	2008	Germany	Cross-sectional	65	Mean (range): 45 (18–80)	60 (92)	Median (range) 3.2 (0.9–3.8)
Author	Reason for amputation^c^	Amputation level^d^	Data collection method	Prosthesis use, *N* (%)	Residual limb pain prevalence (95% CI)^e^	Neuroma prevalence (95% CI)^e^	Risk of bias
Bouteille	I, M, T	H	Medical records	NR		36 (20 to 55)	High
Datta	C, I, M, P, T, V	TE, AE	Self-administered questionnaire	43 (72)	48 (35 to 62)		High
De Lange	T	H	Self-administered questionnaire	NR	32 (20 to 47)		High
Desmond	M, NS, T, WI	H, BE, TE, AE	Self-administered questionnaire	101 (72)	55 (47 to 64)		Low
Ebrahimzadeh	WI	BE, TAE	Interview	NR	24 (9 to 45)		High
Ephraim^a^	M, T, V	BE, AE	Interview	745 (82)	66 (56 to 75)		Low
Fraser	NS	H, BE, AE	Interview	46 (61)	55 (43 to 67)		High
Guo	T	H, BE, AE	Interview	4 (18)	41 (21 to 64)		High
Hanley	C, I, NS, T, V	H, BE, TE, AE	Self-administered questionnaire	59 (57)	71 (61 to 80)		Low
Kooijman	I, M, P, T, V	BE, TE, AE	Self-administered questionnaire	58 (81)	49 (37 to 61)		Low
Lacoux	WI	BE, AE	Interview	NR	100 (91 to 100)	30 (17 to 47)	High
Lans	I, M, NS, T, V	H, BE, AE	Medical records	NR		10 (5 to 16)	Low
O'brien	I, M, NS, T, V, WI	BE, AE	Self-administered questionnaire	NR	64 (50 to 76)		Low
Reiber^b^	WI	H, BE, TE, AE	Self-administered questionnaire	71 (73)	51 (40 to 61)		Low
Resnik	B, D, I, M, NS, T, WI	H, BE, TE, AE	Interview	490 (61)	65 (62 to 69)		Low
Schley	T	H, BE, AE	Self-administered questionnaire	0 (0)	62 (49 to 73)		Low

a Age, sex, follow-up and prosthesis use from cohort of upper and lower extremity amputees combined.

b Follow-up from cohort of upper and lower extremity amputees combined.

c B, Burn; C, congenital; D, diabetes; I, infection; M, malignancy; NS, not specified; P, paralysis of arm; T, trauma; V, vascular; WI, war injury.

d H, Hand; BE, below elbow; TE, through elbow; AE, above elbow.

e 95% Confidence interval (CI) not presented in articles but calculated from sample size and prevalence estimate.

### RLP prevalence

**Figure 2.**
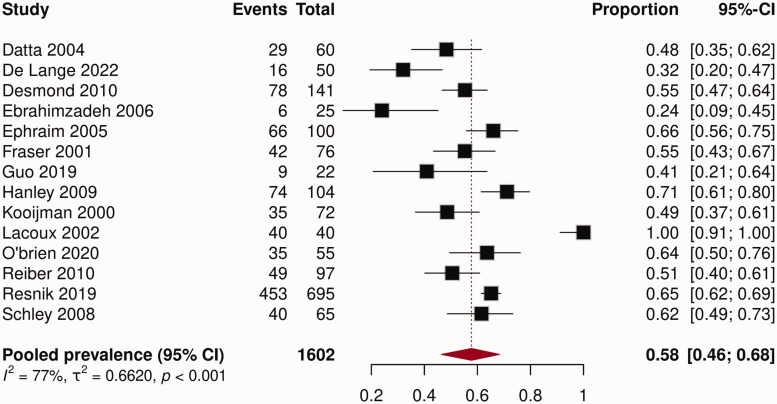
Forest plot prevalence of residual limb pain (RLP).

The prevalence of RLP reported in the included studies ranged from 24 to 100% (Figure 2). The pooled prevalence in this meta-analysis was 58% (95% CI: 46 to 68) with high heterogeneity between studies (*I*^2^ = 77%, *p < *0.001).

Only the subgroup analysis of the characteristic geographic location *(p = *0.004) showed a significant difference (Table 2). Studies conducted in the United States showed a pooled prevalence of 64% (95% CI: 58.1 to 69.0) compared with 51% (95% CI: 44.3 to 57.6) for European studies. No statistically significant differences were found within the subgroups of age (*p = *0.67), reason for amputation (*p = *0.92), data collection method *(p = *0.50) and follow-up period *(p = *0.83). Studies with a mean age of more than 50 years showed a pooled RLP prevalence of 56% (95% CI: 48.3 to 63.8) compared with 62% (95% CI: 35.7 to 83.0) for studies with a mean age of 50 years or less. The pooled RLP prevalences of studies with only traumatic reasons for amputation were 59% (95% CI: 25.6 to 85.5) and 61% (95% CI: 54.6 to 66.3) for studies with other reasons for amputation. Studies using interviews for data collection showed a pooled prevalence of 66% (95% CI: 33.8 to 87.7) compared with 55% (95% CI: 46.7 to 62.0) for studies using self-administered questionnaires. The pooled prevalence of RLP was 57% (95% CI: 39.3 to 73.8) for studies with a follow-up of more than 10 years and 60% (95% CI: 47.4 to 70.9) for studies with a follow-up of 10 years or less. The studies with more than 10 years of follow-up had a mean follow-up period of 31 years, whereas the studies with a follow-up of 10 years or less had a mean follow-up period of 4.8 years. It was not possible to perform a subgroup analysis for amputation level because there were not enough studies with a specific amputation level.

**Table 2.** Subgroup analysis residual limb pain (RLP) prevalence studies

Variable	*N* studies	Prevalence, %	95% CI	*p-*Value^a^
Age				0.67
>50	7	56.2	48.3 to 63.8	
≤50	7	62.3	35.7 to 83.0	
Population				0.004
America	5	63.7	58.1 to 69.0	
Europe	6	51	44.3 to 57.6	
Reason for amputation				0.92
Trauma only	6	58.7	25.6 to 85.5	
Miscellaneous	7	60.6	54.6 to 66.3	
Data collection method				0.50
Interview	6	65.6	33.8 to 87.7	
Self-administered questionnaire	8	54.4	46.7 to 62.0	
Follow-up period				0.83
>10	9	57.4	39.3 to 73.8	
≤10	5	59.8	47.7 to 70.9	

a Calculated by logistic regression analysis using random effects models.

### Prevalence symptomatic neuroma

**Figure 3.**
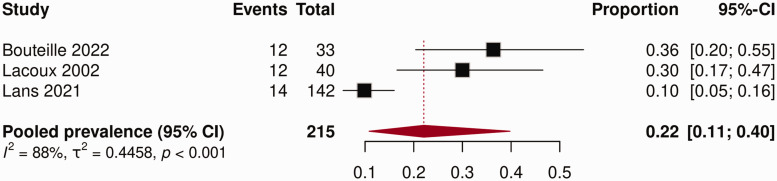
Forest plot prevalence of symptomatic neuroma.

The prevalence of symptomatic neuroma reported in the included studies ranged from 10 to 36% (Figure 3). The pooled prevalence in this meta-analysis was 22% (95% CI: 11 to 40), with high heterogeneity between studies (*I*^2^ = 88%, *p < *0.001). A subgroup analysis of symptomatic neuroma prevalence studies could not be performed owing to the small number of included studies.

## Discussion

The prevalence of RLP varies widely across the included studies with rates ranging from 24 to 100%. The highest RLP prevalence was observed in a cohort of 40 individuals after amputations caused by intentionally inflicted injuries during civil war in Sierra Leone (Lacoux et al., 2002). By structured interviews, participants were asked if they had experienced RLP within the month preceding the interview. This may have resulted in a greater prevalence compared with studies that reported pain at the present time. The two studies reporting the most widely differing prevalences of neuroma occurrence were Lans et al. (2020) (10%) and Bouteille et al. (2022) (36%). Lans et al. (2020) handled nerve ends during amputation either by transection alone or with additional injection of local analgesics, tying off or clipping the nerve end, traction neurectomy, or nerve end implantation into adjacent bone, muscle or soft tissue. The patients of Bouteille et al. (2022) had no additional nerve treatment.

In our subgroup analyses, the prevalence of RLP was lower in studies conducted in Europe than in those in the United States. Healthcare system differences, rehabilitation approaches, variations in prosthetic technology and cultural perspectives towards pain make direct comparison between continents difficult. Socioeconomic factors, such as access to healthcare and prosthetic services, further complicate analysis. These influences make it challenging to isolate specific factors causing the differences in reported RLP prevalence between continents, necessitating cautious interpretation. The European studies lack homogeneity in their representation compared with those in the United States. Owing to the exclusion of studies published in languages other than English, Dutch and German, certain European studies might have been lost.

According to our subgroup analyses, there is no correlation between age, reason for amputation, data collection method or follow-up period and a greater prevalence of RLP. This implies that physicians should be aware of RLP following upper extremity amputations in all patients, regardless of age, length of follow-up or primary reason for amputation. The subgroup analysis evaluated studies with a follow-up of more than 10 years compared with 10 years or less. A more clinically relevant comparison would be a threshold of 2 years, since patients experience changes in nerve regeneration and physical adaptation during this early period. A threshold of 2 years would more accurately represent the difference in RLP prevalence between the early and late stages after amputation. However, owing to the limited number of studies that examined a follow-up period shorter than this limit, it was not feasible to apply this criterion.

Although a higher RLP prevalence may be expected after lower extremity amputations owing to the weightbearing nature, the data indicate that the prevalences are comparable for upper and lower extremities, with incidences of 58 and 59% (95% CI: 51 to 67) (List et al., 2021; Persson and Liedberg, 1982). A systematic review on the prevalence of RLP in military personnel after combat-related amputation reported a pooled prevalence of 61% (Kumar et al., 2024). Our calculated prevalence is comparable with their review, which included individuals with both upper and lower extremity amputations caused by trauma. This implies that the aetiology may not have a major impact on the development of RLP. This is consistent with our subgroup analysis, which found no statistically significant differences in the pooled RLP prevalence between studies on traumatic compared with miscellaneous other aetiologies. A comparison between cohorts with traumatic patients compared with oncological or infectious aetiologies might reveal a difference in prevalence. However, this analysis could not be conducted owing to the absence of studies specifically examining these aetiologies. Evans et al. (2022) performed a systematic review on the prevalence of RLP in patients with amputations resulting from all possible causes, which also included lower extremity amputation and mixed cohorts, but only included prospective studies, and was mostly focused on interventional cohorts. During our literature search, we only included the control groups of the interventional studies. Evans et al. report pooled RLP prevalence at 1 week, 1 month, 3 months, 6 months, 1 year and 2 years after surgery, which were 50, 11, 23, 27, 22 and 24% respectively. For our literature search, a minimum follow-up period of 6 months was applied, and the pooled prevalence was 58%. This notable difference in prevalence may be attributable to the interventions performed and emphasizes the need for such therapies in preventing RLP.

Our study was limited by the small number of included studies on symptomatic neuromas. Only three articles were included in the meta-analysis, and it was not possible to perform neuroma subgroup analyses. Other interesting risk factors such as sex and level of amputation were not examined owing to the absence of specific data. Subgroup analysis indicated higher RLP prevalence in studies conducted within the United States in comparison with Europe. Careful interpretation is necessary owing to data coming from studies that were not comparable between countries, possibly causing reporting bias (Boutron et al., 2022). Notably, there was high heterogeneity among the included studies. The articles had different research designs and methods for data collection. The reported prevalences were mostly based on a binary question regarding presence or absence of RLP without relation to a pain rating, resulting in an undefined clinical impact. Also, included studies used different approaches for data collection. Interviews in person or by telephone are more likely to result in more accurate prevalence than self-administered questionnaires because the interviewer can draw clearer distinctions regarding relevance.

This review revealed a high prevalence of RLP and symptomatic neuromas, highlighting the noteworthy impact of these conditions on patients’ quality of life. It also highlights the need to implement effective postoperative pain management interventions tailored to the individual needs of each patient. Surgeons can use this prevalence data to inform patients about possible outcomes after amputation, set realistic expectations and develop individual treatment strategies with the aim of minimizing discomfort and improving functional outcomes. Continued research and advancement in surgical techniques and prosthetic development will probably improve patient outcomes and reduce the impact of RLP and symptomatic neuromas after upper extremity amputation. Knowledge and awareness about RLP and symptomatic neuroma are important for adequate treatment and prevention aimed at increasing functionality and a better quality of life for upper extremity amputees.

## Supplemental Material

sj-pdf-1-jhs-10.1177_17531934251345368 - Supplemental material for Prevalence of residual limb pain and neuromas after upper extremity amputation: a systematic review and meta-analysis Supplemental material, sj-pdf-1-jhs-10.1177_17531934251345368 for Prevalence of residual limb pain and neuromas after upper extremity amputation: a systematic review and meta-analysis by Emile B List, Vita Klieverik, Enrico Martin, David D Krijgh and J Henk Coert in Journal of Hand Surgery (European Volume)

sj-pdf-2-jhs-10.1177_17531934251345368 - Supplemental material for Prevalence of residual limb pain and neuromas after upper extremity amputation: a systematic review and meta-analysis Supplemental material, sj-pdf-2-jhs-10.1177_17531934251345368 for Prevalence of residual limb pain and neuromas after upper extremity amputation: a systematic review and meta-analysis by Emile B List, Vita Klieverik, Enrico Martin, David D Krijgh and J Henk Coert in Journal of Hand Surgery (European Volume)

sj-pdf-3-jhs-10.1177_17531934251345368 - Supplemental material for Prevalence of residual limb pain and neuromas after upper extremity amputation: a systematic review and meta-analysis Supplemental material, sj-pdf-3-jhs-10.1177_17531934251345368 for Prevalence of residual limb pain and neuromas after upper extremity amputation: a systematic review and meta-analysis by Emile B List, Vita Klieverik, Enrico Martin, David D Krijgh and J Henk Coert in Journal of Hand Surgery (European Volume)

## References

[bibr1-17531934251345368] Arnold DMJ Wilkens SC Coert JH Chen NC Ducic I Eberlin KR. Diagnostic criteria for symptomatic neuroma. Ann Plast Surg. 2019, 82: 420–7. 10.1097/SAP.0000000000001796 30855369

[bibr2-17531934251345368] Bouteille C Saade F El Rifai S Obert L Pluvy I Loisel F. Techniques to prevent symptomatic neuroma in digital amputations. Hand Surg Rehabil. 2022, 41: 234–9. 10.1016/j.hansur.2022.01.003 35074560

[bibr3-17531934251345368] Boutron I Page MJ Higgins JPT Altman DG Lundh A HA. Cochrane Handbook for Systematic Reviews of Interventions, version 6.3, chapter 7. Cochrane, 2022.

[bibr4-17531934251345368] Clarke C Lindsay DR Pyati S Buchheit T. Residual limb pain is not a diagnosis: a proposed algorithm to classify postamputation pain. Clin J Pain. 2013, 29: 551–62. 10.1097/AJP.0b013e318261c9f9 23328317

[bibr5-17531934251345368] Colloca L Ludman T Bouhassira D , et al. Neuropathic pain. Nat Rev Dis Prim. 2017, 3: 17002. 28205574 10.1038/nrdp.2017.2 PMC5371025

[bibr6-17531934251345368] Evans AG Chaker SC Curran GE , et al. Postamputation residual limb pain severity and prevalence: a systematic review and meta-analysis. Plast Surg (Oakville, Ont). 2022, 30: 254–68. 10.1177/22925503211019646 PMC9389065 35990396

[bibr7-17531934251345368] Higgins JPT Thompson SG. Controlling the risk of spurious findings from meta-regression. Stat Med. 2004, 23: 1663–82. 10.1002/sim.1752 15160401

[bibr8-17531934251345368] Hsu E Cohen SP. Postamputation pain: epidemiology, mechanisms, and treatment. J Pain Res. 2013, 6: 121–36. 10.2147/JPR.S32299 PMC3576040 23426608

[bibr9-17531934251345368] Kumar A Soliman N Gan Z , et al. A systematic review of the prevalence of postamputation and chronic neuropathic pain associated with combat injury in military personnel. Pain. 2024, 165: 727–40. 10.1097/j.pain.0000000000003094 PMC10949216 38112578

[bibr10-17531934251345368] Lacoux PA Crombie IK Macrae WA. Pain in traumatic upper limb amputees in Sierra Leone. Pain. 2002, 99: 309–12. 10.1016/s0304-3959(02)00154-9 12237209

[bibr11-17531934251345368] Lans J Hoftiezer Y Lozano-Calderón SA Heng M Valerio IL Eberlin KR. Risk factors for neuropathic pain following major upper extremity amputation. J Reconstr Microsurg. 2020, 37: 413–20. 10.1055/s-0040-1718547 PMC10375759 33058096

[bibr12-17531934251345368] Lee M Guyuron B. Postoperative neuromas. Nerves Nerve Inj. 2015, 2: 99–112.

[bibr13-17531934251345368] List EB Krijgh DD Martin E Coert JH. The prevalence of residual limb pain and symptomatic neuromas following lower extremity amputation: a systematic review and meta-analysis. Pain. 2021, 162: 1906–13. 10.1097/j.pain.0000000000002202 33470746

[bibr14-17531934251345368] Mata DA Ramos MA Bansal N , et al. Prevalence of depression and depressive symptoms among resident physicians a systematic review and meta-analysis. JAMA – J Am Med Assoc. 2015, 314: 2373–83. 10.1001/jama.2015.15845 PMC4866499 26647259

[bibr15-17531934251345368] Moher D Liberati A Tetzlaff J , et al. Preferred reporting items for systematic reviews and meta-analyses: the PRISMA statement. PLoS Med. 2009, 6: e1000097. 10.1371/journal.pmed.1000097 PMC2707599 19621072

[bibr16-17531934251345368] Persson BM Liedberg E. Measurement of maximal end-weight-bearing in lower limb amputees. 1982, 6: 147–51. 10.3109/03093648209166575 7155810

[bibr17-17531934251345368] Rotenstein LS Ramos MA Torre M , et al. Prevalence of depression, depressive symptoms, and suicidal ideation among medical students a systematic review and meta-analysis. JAMA – J Am Med Assoc. 2016, 316: 2214–36. 10.1001/jama.2016.17324 PMC5613659 27923088

[bibr18-17531934251345368] Rotenstein LS Torre M Ramos MA , et al. Prevalence of burnout among physicians a systematic review. JAMA – J Am Med Assoc. 2018, 320: 1131–50. 10.1001/jama.2018.12777 PMC6233645 30326495

[bibr19-17531934251345368] Stang A. Critical evaluation of the Newcastle–Ottawa scale for the assessment of the quality of nonrandomized studies in meta-analyses. Eur J Epidemiol. 2010, 25: 603–5. 10.1007/s10654-010-9491-z 20652370

[bibr20-17531934251345368] Subedi B Grossberg GT. Phantom limb pain: mechanisms and treatment approaches. Pain Res Treat. 2011, 2011: 864605. 22110933 10.1155/2011/864605 PMC3198614

[bibr21-17531934251345368] Ware JE Gandek B. Overview of the SF-36 health survey and the international quality of life assessment (IQOLA) project. J Clin Epidemiol. 1998, 51: 903–12. 10.1016/s0895-4356(98)00081-x 9817107

[bibr22-17531934251345368] Whyte A Niven CA. The illusive phantom: Does primary care meet patient need following limb loss? Disabil Rehabil. 2004, 26: 894–900. 15497918 10.1080/09638280410001708904

