# Supplementary material for: Prevalence of residual limb pain and neuromas after upper extremity amputation: a systematic review and meta-analysis
Source: J Hand Surg Eur Vol. 2025 May 29;50(11):1440–7. doi: 10.1177/17531934251345368 (PMC12686179; doi:10.1177/17531934251345368)
Supplement: sj-pdf-1-jhs-10.1177_17531934251345368 - Supplemental material for Prevalence of residual limb pain and neuromas after upper extremity amputation: a systematic review and meta-analysis [file sj-pdf-1-jhs-10.1177_17531934251345368.pdf]

Appendix S1: Search syntaxes for PubMed and Embase

Pubmed:

(amput*[Title/Abstract] OR disarticulation*[Title/Abstract] OR exarticulation*[Title/Abstract] OR postamputation*[Title/Abstract] OR post amputation*[Title/Abstract] OR limb loss*[Title/Abstract] OR amputation[MeSH Terms] OR amputation stumps[MeSH Terms] OR disarticulation[MeSH Terms]) AND (“stump pain”[Title/Abstract] OR neuroma*[Title/Abstract] OR “stump neuralgia”[Title/Abstract] OR “neuropathic pain”[Title/Abstract] OR “residual limb pain”[Title/Abstract] OR “postamputation pain”[Title/Abstract] OR “pain after amputation”[Title/Abstract] OR neuroma[MeSH Terms])

Embase:

(‘amput*’:ti,ab,kw OR ‘disarticulation*’:ti,ab,kw OR ‘exarticulation*’:ti,ab,kw OR ‘postamputation*’:ti,ab,kw OR ‘post amputation*’:ti,ab,kw OR ‘limb loss*’:ti,ab,kw OR 'amputation'/exp) AND (‘stump pain’:ti,ab,kw OR ‘neuroma*’:ti,ab,kw OR ‘stump neuralgia’:ti,ab,kw OR ‘neuropathic pain’:ti,ab,kw OR ‘residual limb pain’:ti,ab,kw OR ‘postamputation pain’:ti,ab,kw OR ‘pain after amputation’:ti,ab,kw OR 'neuroma'/exp)

Cochrane

(amput*):ti,ab,kw OR (disarticulation*):ti,ab,kw OR (exarticulation*):ti,ab,kw OR (postamputation*):ti,ab,kw OR (post amputation*):ti,ab,kw OR (limb loss*):ti,ab,kw OR MeSH descriptor: [Amputation, Surgical] explode all trees AND (stump pain):ti,ab,kw OR (neuroma*):ti,ab,kw OR (stump neuralgia):ti,ab,kw OR (neuropathic pain):ti,ab,kw OR (residual limb pain):ti,ab,kw OR (postamputation pain):ti,ab,kw OR (pain after amputation):ti,ab,kw OR MeSH descriptor: [Neuroma] explode all trees
