# Supplementary material for: Prevalence of residual limb pain and neuromas after upper extremity amputation: a systematic review and meta-analysis
Source: J Hand Surg Eur Vol. 2025 May 29;50(11):1440–7. doi: 10.1177/17531934251345368 (PMC12686179; doi:10.1177/17531934251345368)
Supplement: sj-pdf-2-jhs-10.1177_17531934251345368 - Supplemental material for Prevalence of residual limb pain and neuromas after upper extremity amputation: a systematic review and meta-analysis [file sj-pdf-2-jhs-10.1177_17531934251345368.pdf]

Appendix S2: Modified Newcastle-Ottawa scoring guide

1. Representativeness of the sample:

    1 point: Population contained multiple reasons and levels* of amputations.

    0 points: Population contained either a single reason, a single level of amputation, or both.

2. Sample size:

    1 point: Sample size was ≥100 participants.

    0 points: Sample size was <100 participants.

3. Non-respondents Comparability:

    1 point: Comparability between respondent and non-respondent characteristics was established, or the response "rate" was 80% or greater.

    0 points: The comparability between respondents and non-respondents was unsatisfactory, the response "rate" was unsatisfactory, or there was no description of the response "rate" or the characteristics of the responders or non-responders."

4. Ascertainment of RLP/SP/Neuroma:

    1 point: Well described and/or validated measurement tool.

    0 points: Poorly described measurement tool of uncertain validity or non-validated single-question screening tool.

5. Quality of descriptive statistics reporting:

    1 point: Reported descriptive statistics to describe the population (e.g., age, sex, follow up) with at least 2 proper measures of dispersion (e.g., mean, standard deviation, range).

    0 points: Descriptive statistics were not reported, were incomplete, or did not include proper measures of dispersion.

The quality assessment tool is used to assess articles on their quality in terms of representativeness, sample size, comparability between respondents and non-respondents, outcome ascertainment and descriptive statistics. The scores range from 0-5 and the studies are classified as low risk of bias (≥3 points) or high risk of bias (<3 points).

* Levels of amputation: Shoulder, above elbow, elbow, below elbow, wrist, below wrist.
