# Supplementary material for: Prevalence of residual limb pain and neuromas after upper extremity amputation: a systematic review and meta-analysis
Source: J Hand Surg Eur Vol. 2025 May 29;50(11):1440–7. doi: 10.1177/17531934251345368 (PMC12686179; doi:10.1177/17531934251345368)
Supplement: sj-pdf-3-jhs-10.1177_17531934251345368 - Supplemental material for Prevalence of residual limb pain and neuromas after upper extremity amputation: a systematic review and meta-analysis [file sj-pdf-3-jhs-10.1177_17531934251345368.pdf]

Online Table S1. Complete quality assessment

Study	Representativeness	Sample size	Comparability	Outcome	Statistics	Total
Bouteille	0	0	0	1	0	1
Datta	1	0	0	0	0	1
De Lange	0	0	0	0	1	1
Desmond	1	1	0	1	1	4
Ebrahimzadeh	0	0	0	1	0	1
Ephraim	1	1	0	1	1	4
Fraser	0	0	0	1	1	2
Guo	0	0	0	1	1	2
Hanley	1	1	0	1	1	4
Kooijman	1	0	1	1	1	4
Lacoux	0	0	0	1	1	2
Lans	1	1	0	1	1	4
O'brien	1	0	0	1	1	3
Reiber	0	1	0	1	1	3
Resnik	1	1	0	1	1	4
Schley	1	0	1	1	1	4
